# Significant role of host sialylated glycans in the infection and spread of severe acute respiratory syndrome coronavirus 2

**DOI:** 10.1371/journal.ppat.1010590

**Published:** 2022-06-14

**Authors:** Wakana Saso, Masako Yamasaki, Shin-ichi Nakakita, Shuetsu Fukushi, Kana Tsuchimoto, Noriyuki Watanabe, Nongluk Sriwilaijaroen, Osamu Kanie, Masamichi Muramatsu, Yoshimasa Takahashi, Tetsuro Matano, Makoto Takeda, Yasuo Suzuki, Koichi Watashi

**Affiliations:** 1 Department of Virology II, National Institute of Infectious Diseases, Tokyo, Japan; 2 The Institute of Medical Science, The University of Tokyo, Tokyo, Japan; 3 AIDS Research Center, National Institute of Infectious Diseases, Tokyo, Japan; 4 Department of Applied Biological Sciences, Tokyo University of Science, Noda, Japan; 5 Department of Functional Glycomics, Life Science Research Center, Kagawa University, Kagawa, Japan; 6 Department of Virology I, National Institute of Infectious Diseases, Tokyo, Japan; 7 Research Center for Drug and Vaccine Development, National Institute of Infectious Diseases, Tokyo, Japan; 8 Department of Preclinical Sciences, Faculty of Medicine, Thammasat University, Pathumthani, Thailand; 9 Department of Medical Biochemistry, School of Pharmaceutical Sciences, University of Shizuoka, Shizuoka, Japan; 10 Micro/Nano Technology Center and Department of Applied Biochemistry, Tokai University, Kanagawa, Japan; 11 Department of Virology III, National Institute of Infectious Diseases, Tokyo, Japan; 12 Institute for Frontier Life and Medical Sciences, Kyoto University, Kyoto, Japan; 13 MIRAI, JST, Saitama, Japan; University of Wisconsin-Madison, UNITED STATES

## Abstract

Severe acute respiratory syndrome coronavirus 2 (SARS-CoV-2) has been transmitted across all over the world, in contrast to the limited epidemic of genetically- and virologically-related SARS-CoV. However, the molecular basis explaining the difference in the virological characteristics among SARS-CoV-2 and SARS-CoV has been poorly defined. Here we identified that host sialoglycans play a significant role in the efficient spread of SARS-CoV-2 infection, while this was not the case with SARS-CoV. SARS-CoV-2 infection was significantly inhibited by α2-6-linked sialic acid-containing compounds, but not by α2–3 analog, in VeroE6/TMPRSS2 cells. The α2-6-linked compound bound to SARS-CoV-2 spike S1 subunit to competitively inhibit SARS-CoV-2 attachment to cells. Enzymatic removal of cell surface sialic acids impaired the interaction between SARS-CoV-2 spike and angiotensin-converting enzyme 2 (ACE2), and suppressed the efficient spread of SARS-CoV-2 infection over time, in contrast to its least effect on SARS-CoV spread. Our study provides a novel molecular basis of SARS-CoV-2 infection which illustrates the distinctive characteristics from SARS-CoV.

## Author summary

SARS-CoV-2, which has been highly transmissible and rapidly spreading worldwide, has caused approximately 458 million confirmed cases of COVID-19 with more than 6 million deaths by March 2022. Here we found that SARS-CoV-2 infection was significantly inhibited by α2-6-linked sialic acid-containing compounds and by depletion of cell surface sialic acid with only a minor effect on SARS-CoV infection. We identified that SARS-CoV-2 spike S1 subunit directly binds to α2-6-linked sialoglycans for efficient attachment to host cell surface. Our finding indicated that host sialoglycans play a significant role in the efficient infection of SARS-CoV-2, which provides a novel understanding of the molecular basis explaining the rapid spread of SARS-CoV-2 over SARS-CoV.

## Introduction

The global spread of severe acute respiratory syndrome coronavirus 2 (SARS-CoV-2), the causative virus of coronavirus disease 2019 (COVID-19), is a public health problem, with an estimated 458 million cases and more than 6 million deaths worldwide by March 2022 [1]. The symptom can range from very mild to severe, including fever, cough, headache, dyspnea, and pneumonia. SARS-CoV-2 is genetically related to SARS-CoV that emerged in 2002. SARS-CoV was highly pathogenic; however, it faded out after having taken intense control measures with a total of about 8,000 infections and 800 deaths [2]. In contrast, SARS-CoV-2 rapidly caused a pandemic with an incredibly high number of worldwide infections, and is still causing social as well as economic damage globally. We previously reported that SARS-CoV-2 achieves its viral load peak in as early as 2.0 days after the onset of its symptoms while that of SARS-CoV peaks at 7.2 days in patients [3]. These evidences highlight the characteristic differences between SARS-CoV-2 and SARS-CoV, which may explain the rapid spread of SARS-CoV-2. The molecular basis underlying the distinct SARS-CoV-2 infection over its related SARS-CoV is not fully understood. It is reported that SARS-CoV-2 receptor binding domain has higher angiotensin-converting enzyme 2 (ACE2) binding affinity than that of SARS-CoV [4,5], which may contribute to the efficient infection of SARS-CoV-2. Other possible explanation for the fast spread of SARS-CoV-2 is that SARS-CoV-2 spike contains furin cleavage site while SARS-CoV does not, thus, allowing SARS-CoV-2 spike to have more chance to be activated for cell entry [5–7]. In this study, we found a novel molecular basis exhibiting the significant role of the host sialic acid in SARS-CoV-2 infection and spread.

The cell entry is the first and essential step in the viral life cycle. The coronavirus mostly enters by the binding between host receptors and the viral spike, which undergoes proteolytic cleavage carried out by host proteases. The activation of spike subsequently promotes viral fusion at the cellular or endosomal membrane, thereby allowing the insertion of the viral genome into the cellular cytoplasm. Human-infecting coronaviruses use different host factors for cell entry. Human coronavirus 229E (HCoV-229E) uses aminopeptidase N as a receptor for viral entry into the host cells [8]. Middle East respiratory syndrome coronavirus (MERS-CoV) uses dipeptidyl peptidase 4 (DPP4) [9]. HCoV-NL63, SARS-CoV, and SARS-CoV-2 recognize the same receptor ACE2 [10–14]. There are several coronaviruses that use sialic acids for cell entry [15]. Of these, three viruses are human-infecting coronaviruses: HCoV-OC43 and HCoV-HKU1 use 9-*O*-acetylated sialic acids as entry receptors [16,17]; whereas MERS-CoV, in addition to DPP4, utilize sialylated glycans during the early attachment phase [18]. While SARS-CoV infection is likely to be independent or minimally dependent on host sialic acid for its attachment [18,19], the role of sialic acid in SARS-CoV-2 infection has been suggested by *in silico* predictions and *in vitro* analysis [20–23]. Only a recent report performed experimental infection studies using viral particles and revealed that sialylated glycans facilitate SARS-CoV-2 infection [24]. These findings highlight the need for further investigation on sialic acid’s role in SARS-CoV-2 infection.

In this study, we screened a series of compounds using SARS-CoV-2 cell culture-based assay and identified α2-6-linked sialic acid-containing compounds to inhibit viral infection. SARS-CoV-2 infection was inhibited by α2-6SLN-lipo-PGA compound in VeroE6/TMPRSS2 and Calu-3 cells. α2-6-linked sialylated glycans targeted SARS-CoV-2 spike S1 subunit to inhibit viral attachment to the host cells. Furthermore, enzymatic removal of endogenous sialic acids on the cell surface by neuraminidase suppressed the spread of SARS-CoV-2 infection, but this was not the case with SARS-CoV. Our study revealed that SARS-CoV-2 utilizes cell surface sialic acid which can support the virus binding to its receptor ACE2. The finding provides novel insight into the molecular basis for the unique characteristics of SARS-CoV-2 infection and spread.

## Results

### α2-6SLN-lipo-PGA inhibited SARS-CoV-2 infection

**Fig 1:**
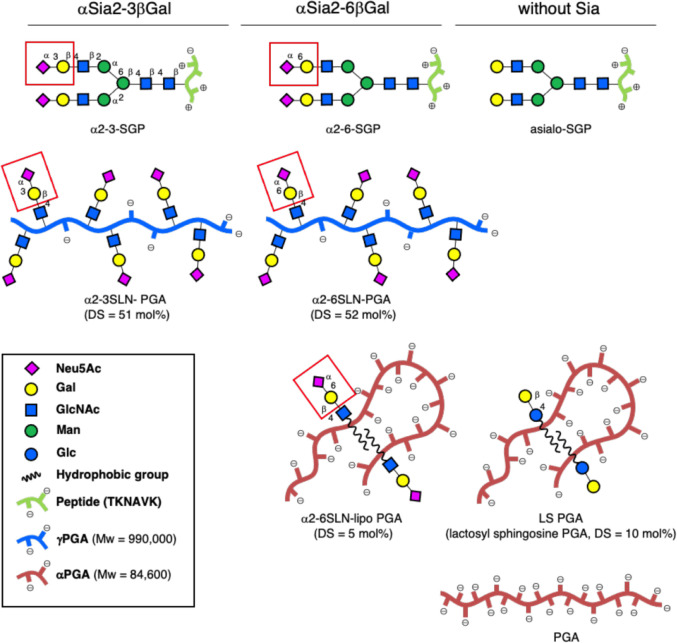
Schematic structures of sialylglyco-polyglutamic acid (PGA) and sialylglycopeptide (SGP). Structures of compounds investigated are shown in a line-column formation. First row: a series of SGP; Second Raw: γ-PGA bearing glycans; Third Row: α-PGA bearing glycans. First column: glycan carrying α-2-3-linked glycosides of sialic acid; Second column: glycan carrying α-2-6-linked glycosides of sialic acid; Third column: compounds without sialic acid. Cartoon presentations of glycans are shown as suggested by the Consortium for Functional Glycomics. Average molecular weights for α- and γ-PGA together with degree of substitution (DS) provide overall structures of polymeric compounds. Polymers carrying “lipo”-functions form self-aggregates.

**Fig 2:**
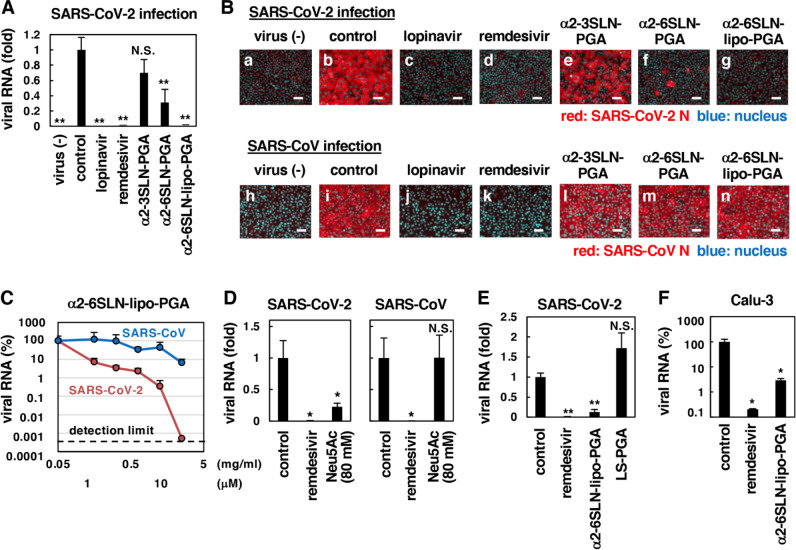
α2-6SLN-lipo-PGA inhibited SARS-CoV-2 infection. **(A, B)** SARS-CoV-2 or SARS-CoV infection assays were performed in the presence or absence of the indicated compounds. SARS-CoV-2 or SARS-CoV infection was determined by detecting SARS-CoV-2 RNA in the culture supernatant (A) and viral N protein in the cells (B). Scale bar 50 μm. Lopinavir, 16 μM; remdesivir, 10 μM; α2-3SLN-PGA, 10 mg/ml; α2-6SLN-PGA, 10 mg/ml; α2-6SLN-lipo-PGA, 1 mg/ml. **(C)** Dose-response curve of α2-6SLN-lipo-PGA upon SARS-CoV-2 (red) or SARS-CoV (blue) infection. Secreted viral RNA was quantified upon treatment with α2-6SLN-lipo-PGA at the concentration as shown. **(D, E)** Anti-SARS-CoV-2 or anti-SARS-CoV activity of *N*-acetylneuraminic acid (Neu5Ac, the component of α2-6SLN-lipo-PGA) and lactosylsphingosine-PGA (LS-PGA, the similar structure with α2-6SLN-lipo-PGA without having sialic acid). Remdesivir, 10 μM; Neu5Ac, 80 mM; α2-6SLN-lipo-PGA, 20 μM; LS-PGA, 20 μM. **(F)** Anti-SARS-CoV-2 activity of indicated compounds in human lung epithelial-derived cell line, Calu-3 cells. Remdesivir, 10 μM; α2-6SLN-lipo-PGA, 10 μM.

By evaluating a series of compounds using SARS-CoV-2 infection cell culture system, we found that α2-6-linked sialic acid-containing compounds inhibited SARS-CoV-2 infection. In this screening we investigated the antiviral activity of compounds using α2-3SLN-PGA [αNeu5Ac-(2–3)-βGal-(1–4)-βGlcNAc-1-aminopentyl-poly-γ-L-glutamic acid], α2-6SLN-PGA [αNeu5Ac-(2–6)-βGal-(1–4)-βGlcNAc-1-aminopentyl-poly-γ-L-glutamic acid], and α2-6SLN-lipo-PGA [αNeu5Ac-(2–6)-βGal-(1–4)-GlcNAc-lipo-poly-α-L-glutamic acid] (see Figs 1 and S1 for structures) [25,26]. VeroE6/TMPRSS2 cells were treated with the compounds for 1 h during inoculation with SARS-CoV-2 at a multiplicity of infection of 0.001. Unbound virus was removed by washing and the cells were treated with compounds for 24 h to assess the viral RNA level in the supernatant as an indicator of released virus particles, cell viability, and viral protein expression (S2A Fig). As shown in Fig 2A, α2-3SLN-PGA slightly, but not significantly, reduced the viral RNA, whereas α2-6SLN-PGA showed significant reduction. α2-6SLN-lipo-PGA exhibited even more reduction of viral RNA along with the positive control drugs lopinavir and remdesivir without showing cytotoxicity (S2B Fig). α2-6SLN-PGA and α2-6SLN-lipo-PGA consistently reduced SARS-CoV-2 N protein expression while α2-3SLN-PGA treatment showed very little decrease as compared to the control (Fig 2B, panel e-g). Considering these results, we focused on α2-6SLN-lipo-PGA for further analysis. α2-6SLN-lipo-PGA reduced SARS-CoV-2 RNA in a concentration-dependent manner to less than 0.001% of the untreated control infections (Fig 2C, red) without cytotoxicity (S2C Fig). In contrast, α2-6SLN-lipo-PGA reduced the SARS-CoV infection marginally to around 10% at most (Fig 2C, blue). An immunofluorescence detection of SARS-CoV N protein also showed only a little or no effect of α2-6SLN-lipo-PGA compound (Fig 2B, panel n), which almost completely eliminated the SARS-CoV-2 N protein in the same infection assay (Fig 2B, panel g). We then investigated the antiviral activity of the component of α2-6SLN-lipo-PGA. Independent treatment of *N*-acetylneuraminic acid (Neu5Ac, see Fig 1 and S1 for the structure), which is the sialic acid in α2-6SLN-lipo-PGA, exhibited antiviral activity (Fig 2D, left), while this was not the case with SARS-CoV infection (Fig 2D, right). We further confirmed that methyl α-Neu5Ac and α2–6 linked triose (α2–6 sialyllactose) inhibited SARS-CoV-2 infection (S2D and S2E Fig). On the contrary, lactosylsphingosine-PGA [βGal-(1–4)-βGlc-1-sphingosine-poly-α-L-glutamic acid] (LS-PGA, see Figs 1 and S1 for the structure), which is composed of similar structure to α2-6SLN-lipo-PGA without having sialic acid, did not reduce the viral RNA (Fig 2E). The data indicates that the sialic acid, and not the SLN-lipo-PGA backbone, is responsible for the antiviral activity of α2-6SLN-lipo-PGA. We also confirmed that α2-6SLN-lipo-PGA showed a log-scale reduction in SARS-CoV-2 infection in a human-derived lung epithelial cell line Calu-3 cells (Fig 2F).

### α2-6SLN-lipo-PGA inhibited SARS-CoV-2 attachment

**Fig 3:**
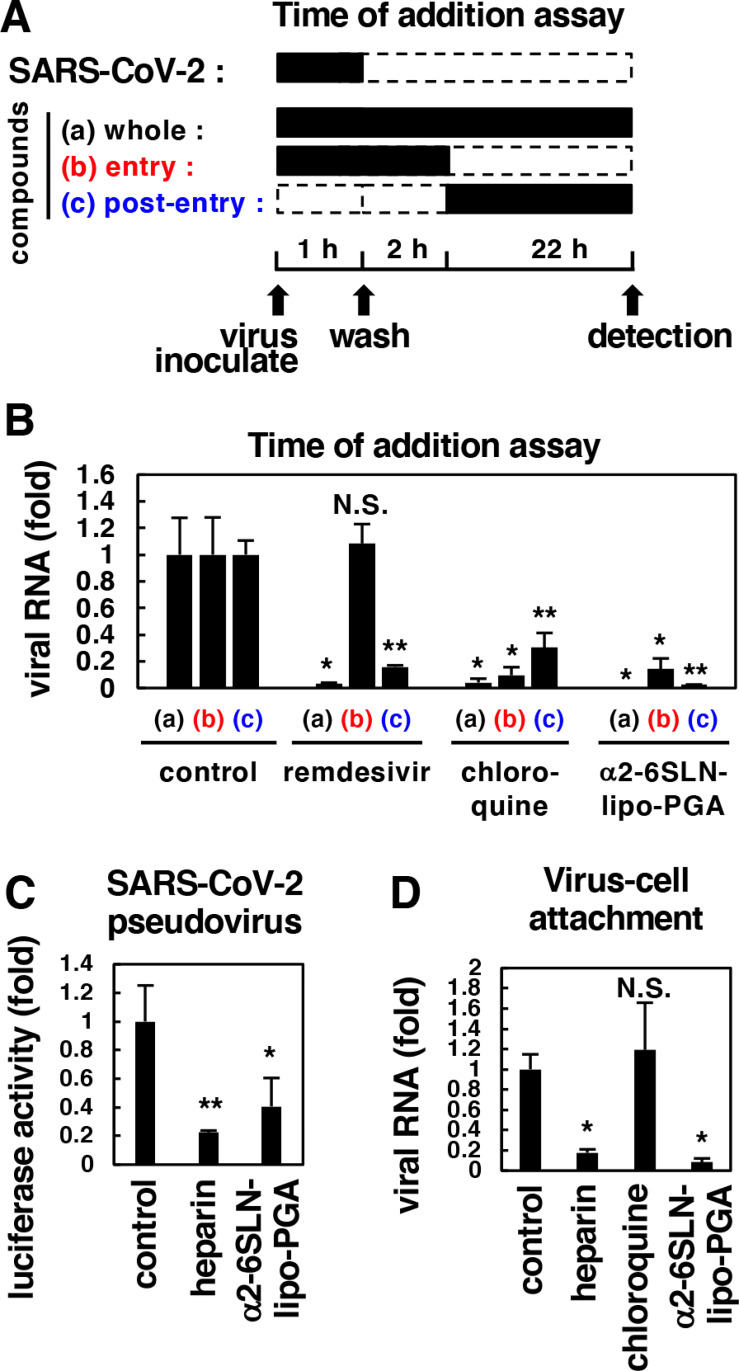
α2-6SLN-lipo-PGA inhibited SARS-CoV-2 attachment. **(A)** Schematic representation of the schedule for treating VeroE6/TMPRSS2 cells with compounds and SARS-CoV-2 in time of addition analysis. Black and white boxes indicate the periods with and without treatment, respectively. **(B)** The antiviral activities of indicated compounds under treatment protocol as shown in (A) are estimated by quantifying the levels of secreted viral RNA at 24 h post-inoculation. Remdesivir, 8 μM; chloroquine, 8 μM; α2-6SLN-lipo-PGA, 20 μM. **(C)** SARS-CoV-2 attachment/entry was evaluated using SARS-CoV-2 pseudovirus. VeroE6/TMPRSS2 cells were inoculated with SARS-CoV-2 pseudovirus in the presence or absence of compounds, and at 24 h post-inoculation, cells were lysed and assessed for luciferase activity generated by SARS-CoV-2 pseudovirus infection. Heparin, 50 U/ml; α2-6SLN-lipo-PGA, 10 μM. **(D)** Virus-cell attachment assay. VeroE6/TMPRSS2 cells were incubated with viruses in the presence or absence of the indicated compounds for 5 min at 4°C to allow virus-cell attachment. After extensive washing, cells were lysed and cell-attached viral RNA was quantified. Heparin, 50 U/ml; chroloquine, 100 μM; α2-6SLN-lipo-PGA, 100 μM.

To investigate which step in the viral life cycle was blocked by α2-6SLN-lipo-PGA, we performed a time of addition assay (Fig 3A). The antiviral activity of compounds was measured under three different conditions: (a, whole) compounds present throughout the experiment, (b, entry) compounds were treated for 1 h during viral inoculation and additional 2 h and then removed, and (c, post-entry) compounds were added at 2 h after washing free viruses and remained throughout the experiment. As shown in Fig 3B, SARS-CoV-2 replication inhibitor remdesivir treated at (a) whole and (c) post-entry showed significant viral RNA reduction without showing significant effect on viral entry (b). Chloroquine, a known SARS-CoV-2 entry inhibitor, was confirmed to show antiviral activity in the early stages of infection (Fig 3B, Chloroquine, b). Since this assay allows multiple rounds of re-infection, entry inhibitors can show antiviral effects when added at (c) post-entry (Fig 3B, Chloroquine, c). Since α2-6SLN-lipo-PGA treatment inhibited viral infection at all three conditions (Fig 3B, α2-6SLN-lipo-PGA, a-c), α2-6SLN-lipo-PGA was suggested to inhibit the viral entry process. We further confirmed the result using SARS-CoV-2 pseudovirus; which consists of vesicular stomatitis virus-based pseudovirus carrying SARS-CoV-2 spike, to evaluate SARS-CoV-2 spike-dependent cell entry. As Fig 3C shows, SARS-CoV-2 pseudovirus infection was significantly inhibited by the treatment with α2-6SLN-lipo-PGA, as well as heparin, which inhibits cell attachment through heparan sulfate proteoglycan in multiple viruses including SARS-CoV-2 [27,28]. We then examined the effect of the compound on viral attachment to the host cell surface, by incubating the cells with SARS-CoV-2 at 4°C to allow virus-cell attachment but not the following process, and quantifying viral RNA attached on the cell surface (Fig 3D). Heparin significantly inhibited SARS-CoV-2 attachment to cells, while chloroquine, which targets intracellular trafficking pathways, had no effect on viral attachment. α2-6SLN-lipo-PGA clearly blocked SARS-CoV-2 particle attachment. These data suggest that α2-6SLN-lipo-PGA inhibits SARS-CoV-2 attachment to host cells.

### α2-6SLN-lipo-PGA targets SARS-CoV-2 particles to inhibit viral attachment

**Fig 4:**
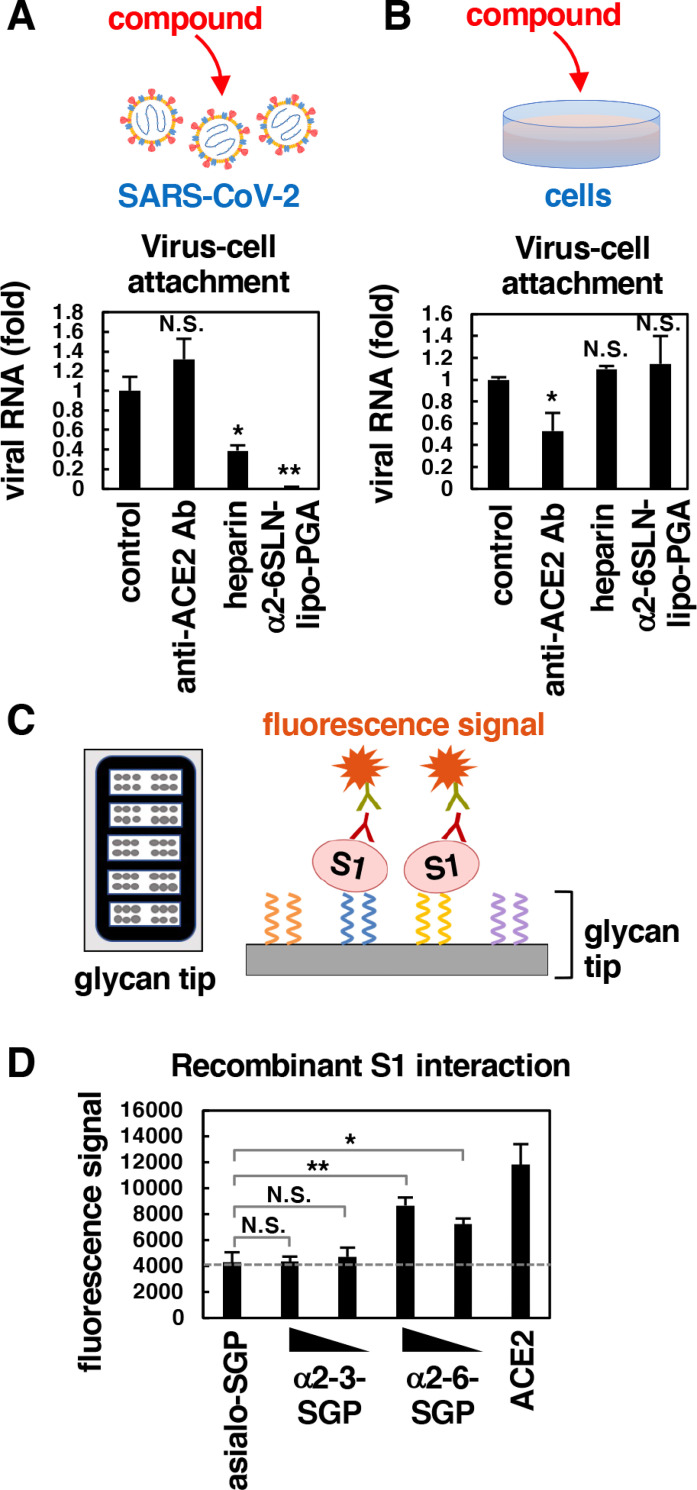
α2-6-linked sialosides interacted with S1 subunit of SARS-CoV-2 spike protein. **(A)** The attachment of compound-pretreated virus to cells. SARS-CoV-2 particles were pretreated with the compounds at 37°C for 60 min, then the free compounds were removed by ultrafiltration. The prepared viruses were treated to VeroE6/TMPRSS2 cells in the absence of compounds at 4°C for 30 min to examine virus-cell attachment. **(B)** The viral attachment to compound-pretreated cells. The indicated compounds were treated to VeroE6/TMPRSS2 cells at 37°C for 30 min and were then washed out extensively. The prepared cells were incubated with SARS-CoV-2 in the absence of compounds at 4°C for 30 min to examine virus-cell attachment. (A, B) Anti-ACE2 antibody, 100 μg/ml; heparin, 10 U/ml; α2-6SLN-lipo-PGA, 10 μM. **(C)** Schematic representation of glycan array. Glycan tip immobilized with sialylglycopeptides on a slide glass was incubated with recombinant SARS-CoV-2 S1 at room temperature for 1 h, and then the fluorescence signal was detected. Anti-SARS-CoV-2 S1 antibody was used as primary antibody, and Cy3-conjugated anti-rabbit IgG was used as secondary antibody. **(D)** The fluorescent response of the glycan array due to the interaction between recombinant SARS-CoV-2 S1 (aa 16–685, Sino Biological) and indicated protein or glycans. The amount of glycan on the neoglycoproteins were 10.8 mol (α2-6-SGP), 10.5 mol (α2-3-SGP) and 10.8 mol (asialo-SGP) of glycan per 1 mol of BSA. The dashed line indicates the nonspecific response level derived from negative control asialo-SGP. Asialo-SGP, 0.7 mg/ml; α2-3-SGP, 0.1 and 1 mg/ml; α2-6-SGP, 0.1 and 1 mg/ml; recombinant ACE2, 0.1 mg/ml.

We then examined whether α2-6SLN-lipo-PGA targeted SARS-CoV-2 particles or host cells to inhibit viral infection. At first, to determine whether α2-6SLN-lipo-PGA targeted viral particles, we prepared “compound-treated SARS-CoV-2 particles” by incubating SARS-CoV-2 with or without compounds for 1 h and then removing unbound compounds by ultrafiltration. The capacity of the prepared virus for cell attachment was examined by treating VeroE6/TMPRSS2 cells for 30 min at 4°C and quantifying the attached virus. The viral attachment was greatly reduced by the treatment with α2-6SLN-lipo-PGA, as well as heparin that targets viral particle, but not with anti-ACE2 antibody (Fig 4A). To determine whether α2-6SLN-lipo-PGA targets host cells, we prepared “compound-treated cells” by incubating VeroE6/TMPRSS2 cells with compounds for 30 min. After washing out free compounds, the cells were inoculated with virus for 30 min at 4°C in the absence of compounds and attached viral particles were detected. Cell susceptibility to SARS-CoV-2 attachment was significantly reduced when treated with anti-ACE2 antibody, but not with α2-6SLN-lipo-PGA (Fig 4B). These results suggest that α2-6SLN-lipo-PGA targets SARS-CoV-2 particles to inhibit viral infection.

### α2-6-linked sialosides interacted with the S1 subunit of SARS-CoV-2 spike protein

We further analyzed the direct interaction between SARS-CoV-2 spike and sialic acids *in vitro*. Spike protein is divided into S1 and S2 subunits that are responsible for viral attachment to cell and virus-cell membrane fusion, respectively. Since α2-6-linked sialosides inhibited viral attachment to host cells (Fig 3D), we hypothesized that α2–6 sialylated glycans might target SARS-CoV-2 S1 subunit; we tested this hypothesis by glycan array. ACE2 (positive control), asialo-SGP (negative control), α2-3-SGP, and α2-6-SGP (see Fig 1 for the structure) were immobilized on a glass slides and were incubated with recombinant SARS-CoV-2 S1 protein (aa 16–685) (Fig 4C). As Fig 4D shows, incubated recombinant S1 interacted with ACE2 and yielded the fluorescence signal, confirming that the assay is working properly, whereas asialo-SGP, which does not contain sialosides, showed much less response. α2-6-SGP yielded significant responses in a dose-dependent manner, while α2-3-SGP exhibited as much response as the negative control asialo-SGP, regardless of the concentration. This result indicates that α2-6-linked sialylated glycans directly interact with the S1 subunit of SARS-CoV-2 spike.

### Host sialic acid contributed to the efficient spread of SARS-CoV-2 infection

**Fig 5:**
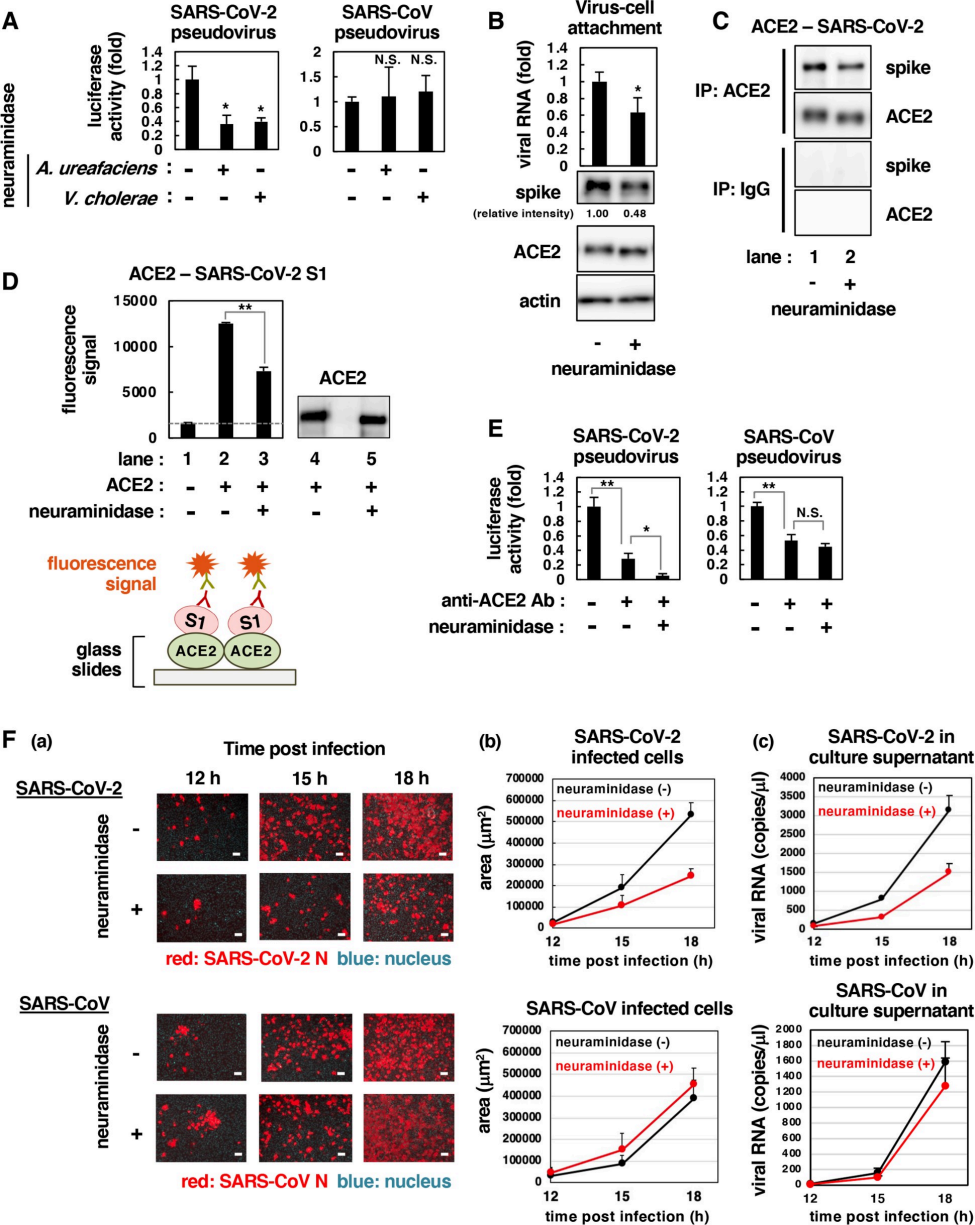
Host sialic acid contributed to the efficient spread of SARS-CoV-2 infection. **(A)** SARS-CoV-2 or SARS-CoV pseudovirus infection to neuraminidase-treated cells. VeroE6/TMPRSS2 cells were pretreated with or without indicated neuraminidases for 2 h at 37°C, followed by inoculation with SARS-CoV-2 or SARS-CoV pseudovirus. At 24 h post-inoculation, cells were lysed and luciferase activity was quantified. **(B)** SARS-CoV-2 attachment on neuraminidase-treated cells. VeroE6/TMPRSS2 cells were treated with or without neuraminidase for 2 h at 37°C, then the cells were inoculated with SARS-CoV-2 for 1 h at 4°C. After washing unbound viruses, the cells were lysed and attached viral RNA was detected. The attached viral spike protein and endogenous protein expression of ACE2 and actin in VeroE6/TMPRSS2 cells was determined by immunoblotting. The number under the blot shows the relative intensity (fold) of the band measured using ImageJ program. **(C)** The interaction between ACE2 and SARS-CoV-2 spike under pretreatment with neuraminidase. VeroE6/TMPRSS2 cells were treated with or without neuraminidase for 2 h at 37°C, then were inoculated with SARS-CoV-2 for 1 h at 4°C. After washing unbound viruses, the cells were harvested to perform immunoprecipitation with anti-ACE2 antibody (IP: ACE2) or normal mouse IgG as a negative control (IP: IgG). Viral spike protein and ACE2 in the precipitates were detected by immunoblotting. **(D)** The effect of sialic acid on ACE2 for the interaction with SARS-CoV-2 spike S1. The fluorescent response due to the interaction between recombinant SARS-CoV-2 spike S1 and recombinant ACE2 protein was detected. Recombinant ACE2 proteins were incubated with or without 2 U/ml neuraminidase (*Arthrobacter ureafaciens*) for 2 h at 37°C, and each ACE2 sample were immobilized on a glass slide to analyze the interaction with SARS-CoV-2 spike S1 protein. The dashed line indicates the nonspecific response level of protein-free glass slide. The schematic representation of the assay is shown under the data. The removal of sialic acid on ACE2 by treatment with neuraminidase was confirmed by immunoblotting. **(E)** Combination treatment with anti-ACE2 antibody and neuraminidase was performed using SARS-CoV-2 and SARS-CoV pseudovirus. **(F)** The spread of SARS-CoV-2 and SARS-CoV infection was examined by (a) immunofluorescent observation of infected cells (scale bar 50 μm), (b) quantification of infected area and (c) detection of viral RNA in culture supernatant. (A-F) Neuraminidase from *Vibrio cholerae*, 40 mU/ml (A); from *Arthrobacter ureafaciens*, 100 mU/ml (A, E), 200 mU/ml (B, C) and 2 U/ml (D, F); anti-ACE2 antibody, 40 μg/ml (E, left) and 7 μg/ml (E, right).

The use of cell surface sialic acid by SARS-CoV-2 was examined by the neuraminidase-mediated removal of cell surface sialic acid. Upon preincubating VeroE6/TMPRSS2 cells with neuraminidase from either *Arthrobacter ureafaciens* or *Vibrio cholerae*, SARS-CoV-2 pseudovirus infection significantly reduced (Fig 5A, left). The removal of sialic acid from VeroE6/TMPRSS2 cells by treatment with neuraminidase was confirmed by lectin staining (S3 Fig). In contrast, infection of SARS-CoV pseudovirus was not affected by neuraminidase treatment (Fig 5A, right). Consistently, removal of sialic acid reduced SARS-CoV-2 attachment to the cell surface, as monitored by detecting viral RNA and spike protein derived from attached viral particles, although the cell expression of ACE2 was not decreased upon neuraminidase treatment (Fig 5B). We then examined the role of sialic acid in virus-ACE2 binding by incubating neuraminidase-treated or untreated cells with SARS-CoV-2 at 4°C to examine spike-ACE2 binding by coimmunoprecipitation assay: SARS-CoV-2 spike was copurified with endogenous ACE2 in intact cells (Fig 5C, lane 1), whereas this ACE2-coprecipitated SARS-CoV-2 spike was impaired by depletion of sialic acids upon neuraminidase treatment (Fig 5C, lane 2). Moreover, we found that the interaction of ACE2 and SARS-CoV-2 spike S1 was impaired when mammalian cell-derived recombinant ACE2 was pretreated with neuraminidase (Fig 5D, lanes 2, 3). The removal of sialic acid on the recombinant ACE2 by neuraminidase was confirmed by mobility shift of the band in gel by immunoblotting (Fig 5D, lanes 4, 5). Thus, host sialic acid on the cell surface facilitates the binding of SARS-CoV-2 S1 and ACE2. Fig 5E indicates that dual-inhibition of ACE2 and sialic acid had more inhibitory effect than mono-treatment of anti-ACE2 antibody in SARS-CoV-2 pseudovirus infection, which was not the case with SARS-CoV pseudovirus infection. Finally, time-dependent spread of SARS-CoV-2 and SARS-CoV infection was examined. The spread of SARS-CoV-2 infection detected by SARS-CoV-2 N protein over time post infection was more moderate upon neuraminidase treatment (Fig 5F-a, upper), which was quantitatively demonstrated by detecting virus infected area (Fig 5F-b, upper) or viral RNA in culture supernatant (Fig 5F-c). In contrast, the spread of SARS-CoV infection was readily observed irrespective of the removal of sialic acid (Fig 5F-a, b, c, lower). These results suggest that SARS-CoV-2 utilizes sialic acid to facilitate cell attachment and binding to ACE2, which leads to an efficient spread of the viral infection.

## Discussion

In this study, using cell culture-based infection assay, we identified that α2-6-linked sialic acid-containing compounds inhibited SARS-CoV-2 infection, but not SARS-CoV. It was revealed that the compound targeted virus particles to inhibit viral attachment to cells. *In vitro* analysis using recombinant SARS-CoV-2 spike protein and sialylglycopeptides consistently showed that α2-6-linked sialylated glycan directly interacted with the S1 subunit of SARS-CoV-2 spike protein. We demonstrated that depletion of sialic acids from the cell surface using neuraminidase impaired the interaction between SARS-CoV-2 spike and ACE2, and eventually suppressed the spread of SARS-CoV-2 infection, while SARS-CoV spread was independent of the removal of sialic acid. Our data clearly indicated the distinct difference in the infection dependency on sialic acids between SARS-CoV-2 and SARS-CoV. These findings suggest that host sialosides play an important role in the efficient infection of SARS-CoV-2.

The sialic acid-binding capacity is one of the many determinants of biological diversity in the family of coronavirus. Several coronaviruses have been reported to interact with sialic acids, including HCoV-OC43, HCoV-HKU1, bovine CoV (BCoV), mouse hepatitis virus, MERS-CoV, transmissible gastroenteritis virus, and infectious bronchitis virus [16–18,29,30]. For HCoV-OC43 and HCoV-HKU1, 9-*O*-acetylated-Sia (Neu5,9Ac_2_) is the sole receptor, in which the binding critically determines the cell entry of the virus. In contrast, the sialic acid use of SARS-CoV-2 is more similar to that of MERS-CoV, which involves binding to the cell surface of sialylated glycans contributing to the efficiency of binding to their entry receptor DPP4. Fantini et al. [31] proposed a dual receptor/attachment model for SARS-CoV-2 with the spike protein involved in ACE2 receptor recognition and finding sialylated sphingoglycolipid (ganglioside)-rich landing area (lipid rafts) at the cell surface. Considering our result that depletion of cell surface sialic acids reduces the capacity of cells for the binding of SARS-CoV-2 particles with the impairment of ACE2 interaction to the viral spike protein, the interaction of SARS-CoV-2 with sialic acids may be responsible for the early contact with host cells, which can serve in concentrating the viruses on the surface of target cells, facilitating their binding to high-affinity receptor ACE2.

The genome sequence homology of SARS-CoV-2 exhibits 79.6% similarity with SARS-CoV, and they share the same receptor ACE2 for their entry into host cells [12–14]. Despite the similarities, our data demonstrated one of their differences that SARS-CoV-2 has the ability to use host cell sialosides while SARS-CoV does not. The N-terminal domain (NTD) of spike protein is responsible for glycan binding in other coronaviruses such as BCoV, HCoV-OC43, and MERS-CoV. The NTD sequence of SARS-CoV-2 and SARS-CoV show only 53.5% of homology, while full-length spike proteins share almost 76% identities [19]. Seyran et al. [20] proposed the presence of the distinct flat and non-sunken sialic acid-binding domain in SARS-CoV-2 S1-NTD which enables faster viral surfing over the epithelium. This promotes faster viral movement to ACE2 receptor compared to other human coronaviruses such as SARS-CoV. Moreover, the structure-based sequence comparison of the spike protein of SARS-CoV, SARS-CoV-2, and MERS-CoV identified that SARS-CoV-2 S1-NTD contains structural regions reminiscent of MERS-CoV sialoside binding pockets [21,22]. The sialic acid binding of recombinant SARS-CoV-2 spike was experimentally investigated by Baker et al. [23] using gold nanoparticles bearing sialic acid, which demonstrated that αNeu5Ac is a ligand for spike glycoprotein, although they did not examine the function of αNeu5Ac in virus infection. Nguyen et al. [24] presented a new idea that receptor binding domain (RBD) of SARS-CoV-2 spike is involved in the recognition of sialylated glycans. They performed *in vitro* glycan library screening against SARS-CoV-2 RBD and S protein, which revealed that several sialic acid-containing structures are recognized. The usage of glycolipids by SARS-CoV-2 was confirmed by pharmacological abrogation in viral infection. These findings are consistent with our result that SARS-CoV-2 S1 interact with sialic acid-containing molecules, which supports viral entry into host cells.

Sialic acids are widely distributed in human tissues and sialylated glycans are present in glycoproteins (*N*-glycans, *O*-glycans), glycosphingolipids (gangliosides), certain GPI anchors, polysialic acids, and others [32,33]. Most sialic acid-binding viruses appear to bind to non-reducing terminal sialic acid linked to a penultimate sugar residue galactose (Gal) via an α2–6 or α2–3 linkage [34–37]. HCoV-OC43 prefers Neu5,9Ac_2_ linked to Gal via α2–6 over α2–3 linkage [38]. MERS-CoV binds to sialylated glycans containing α2-3-linked sialic acids rather than α2–6, which correlates with the differential distribution of α2–3 or α2-6-linked sialic acids in the target cells of the hosts [18,39]. Our study demonstrated that SARS-CoV-2 infection was effectively inhibited by α2-6SLN-lipo-PGA and α2-6SLN-PGA, and SARS-CoV-2 spike protein was preferentially bound to α2-6Neu5Ac-containing SGP. The sialylated glycans that were used in this study possessed α2-6-linked sialyllactosamine type 2 sequence [αNeu5Ac-(2–6)-βGal-(1–4)-βGlcNAc-1-]. Glycans possessing sialyllactosamine type 2 sequences are ubiquitously distributed in human respiratory tract with different sialic acid linkages and lactosamine repeats [40–44]. α2–6 sialylated glycans are predominantly expressed in a human’s upper respiratory tissues, while the lower respiratory tract including lung and bronchi contains a mixture of α2–3 and α2–6 linkage. Interestingly, ACE2 expression in the respiratory tissue is limited as compare to other organs such as the heart or kidney [45,46]. Above results may indicate that glycans consisting of α2-6-linked sialyllactosamine type 2 sugar chains at upper and lower respiratory tissues mediate efficient SARS-CoV-2 spread in the limited ACE2-expressing cells. This may consequently result in the short interval until the viral load peaks in patients. Also, SARS-CoV-2 interactions with sialylated glycans at upper respiratory tract may contribute to the efficient transmission to other organisms by respiratory fluids, which possibly caused the rapid spread of SARS-CoV-2 throughout the world as compared to the SARS-CoV. However, precise preference of SARS-CoV-2 for various types of sialoside requires further analysis in future studies.

As the viral entry process is essential for initiation, spread, and maintenance of infection, this step is an attractive target for the development of antiviral agents. Our finding, that SARS-CoV-2 utilizes sialic acids for its entry, provides novel insight into understanding the molecular mechanisms of SARS-CoV-2 infection and the role of sialylated glycans in viral entry as well as the developing antiviral strategies.

## Materials and methods

### Cell culture

VeroE6 cells overexpressing transmembrane protease, serine 2 (TMPRSS2) [47,48] were cultured in Dulbecco’s modified Eagle’s medium (Life Technologies) supplemented with 10% fetal bovine serum (FBS; Cell Culture Bioscience), 100 units/mL penicillin, 100 μg/mL streptomycin, 10 mM HEPES (pH 7.4), and 1 mg/mL G418 (Nacalai) at 37°C in 5% CO_2_. During the infection assay, 10% FBS was replaced with 2% FBS and G418 removed. Calu-3 cells were cultured in the above medium without G418.

### Reagents

Remdesivir was purchased from Chemscene, lopinavir from Selleck, chloroquine from Nacalai Tesque, heparin from Mochida Pharmaceutical, neuraminidase from Roche and Nacalai Tesque, anti-ACE2 antibody from R&D Systems, glycopeptides (α2-6-SGP, α2-3-SGP, and asialo-SGP) from Fushimi Pharmaceutical, 2-*O*-methyl α-D-*N*-acetylneuraminic acid (Me-α-Neu5Ac) and α2–6 sialyllactose sodium salt from Nagara Science, and recombinant SARS-CoV-2 spike S1 and ACE2 proteins from Sino Biological.

### Compound synthesis

αNeu5Ac-(2–3)-βGal-(1–4)-βGlcNAc-1-aminopentyl-poly-γ-L-glutamic acid (α2-3SLN-PGA) and αNeu5Ac-(2–6)-βGal-(1–4)-βGlcNAc-1-aminopentyl-poly-γ-L-glutamic acid (α2-6SLN-PGA) used in this study were synthesized as described previously [49]. αNeu5Ac-(2–6)-βGal-(1–4)-GlcNAc-lipo-poly-α-L-glutamic acid (α2-6SLN-lipo-PGA) was prepared in-house according to a procedure previously reported [25]. The concept of the polymer design is important where negatively charged poly-Glu backbone with or without hydrophilic glycans has random coil structure, however, introduction of lipophilic tail prompts its form more globular overall by intra-hydrophobic interactions. This is advantageous for presentation of glycan part to outer layer of the globule. Since the degree of substitution (DS) is low, such hydrophobic interaction is rather unstable. Thus, on binding with a viral protein, the polymer structure become unstable, and the exposed lipid tails interact with nearby hydrophobic environment possibly insertion into a hydrophobic patch of a protein or into lipid bilayer. The lipid chain connected to Lys in α2-6SLN-lipo-PGA has been shown to mimic sphingosine. The content of sialic acid moiety (approximately 5 mol%) was estimated according to the ^1^H-nuclear magnetic resonance analysis (Advance 500 spectrometer, Bruker Biospin). βGal-(1–4)-βGlc-1-sphingosine-poly-α-L-glutamic acid (LS-PGA) was prepared in-house according to a procedure similar to one reported [50]. Briefly, a solution of LS (0.13 mg, 0.2 mmol) and PGA sodium salt (average mol. wt. 84,600, 3.0 mg, 10 equiv. as L-Glu) dissolved in 15 mL of THF-H_2_O (2:1) was added 4-(4,6-dimethoxy-1,3,5-triazin-2-yl)-4-methylmorpholinium chloride (DMT-MM, 6.0 mg, 20 mmol), and the resulting mixture was stirred overnight at 40°C. After the disappearance of starting amine was confirmed by TLC (EtOAc/MeOH/H_2_O = 2:1:1), the reaction mixture was concentrated to give a residue, which was then dissolved in 5% NaHCO_3_, loaded onto a G-25 column, and eluted with MeOH to afford LS-PGA. The degree of substitution of LS in the polymer was about 10 mol%.

### SARS-CoV-2 and SARS-CoV infection assay

VeroE6/TMPRSS2 cells were seeded in 96 well plates a day before virus infection. The cells were inoculated with SARS-CoV-2 Wk-521 strain, a clinical strain isolated from a COVID-19 patient [47], or SARS-CoV Frankfurt strain at a multiplicity of infection (MOI) of 0.001 or 0.003 for 1 h and the unbound virus was removed by washing. Cells were cultured for 24 h prior to measuring the extracellular viral RNA or detecting viral encoded N protein. Compounds were added during virus inoculation (1 h) and replenished after washing (24 h). Infection assay with Calu-3 cells was performed by incubation with virus at a MOI of 0.1 for 3 h. Extracellular viral RNA was detected at 72 h post-inoculation. For the calculation of MOI, viral titers (50% tissue culture infective dose: TCID_50_) were determined by Behrens-Karber method [51].

### Quantification of viral RNA

Viral RNA was extracted with a QIAamp Viral RNA Mini Kit (QIAGEN), RNeasy Mini Kit (QIAGEN) or MagMAX Viral/Pathogen II Nucleic Acid Isolation Kit (Thermo Fisher Scientific) and quantified by real time RT-PCR analysis with a one-step qRT-PCR kit (THUNDERBIRD Probe One-step qRT-PCR kit, TOYOBO) using 5’-ACAGGTACGTTAATAGTTAATAGCGT-3’, 5’- ATATTGCAGCAGTACGCACACA-3’, and 5’-FAM-ACACTAGCCATCCTTACTGCGCTTCG-TAMRA-3’ (SARS-CoV-2 and SARS-CoV relative quantification) [52]; 5’-AAATTTTGGGGAVVAGGAAC-3’, 5’-TGGCAGCTGTGTAGGTCAAC-3’, and 5’-FAM-ATGTCGCGCATTGGCATGGA-TAMRA-3’ (SARS-CoV-2 absolute quantification); 5’-GAACAAACCCAAGGAAATTT-3’, 5’-GGATCTTTGTCATCCAATTT-3’, and 5’-FAM-ATGTCGCGCATTGGCATGGA-TAMRA-3’ (SARS-CoV absolute quantification).

### Cell viability

At 24 h post infection, the same time point when culture supernatant was collected to measure extracellular viral RNA, cells were washed with PBS and fixed with 4% paraformaldehyde. Then the cells were stained with DAPI. Cell viability was determined by the quantification of survived cell numbers by counting the number of detected nucleus with three cell culture wells (one microscopic field in each well) in a 96 well plate using a high content imaging analyzer ImageXpress Micro Confocal (Molecular Device) [53,54].

### Indirect immunofluorescence analysis

The cells were fixed with 4% paraformaldehyde and permeabilized with 0.3% Triton X-100. To detect viral protein expression, the cells were treated with a rabbit anti-SARS-CoV/SARS-CoV-2 N antibody [55] as a primary antibody, and then incubated with Alexa594-conjugated secondary antibody and DAPI. N-positive cells (Fig 5F-b) were quantified by calculating fluorescent stained area of three cell culture wells (one microscopic field in each well) in a 96 well plate using hybrid cell count system in BZ-X Analyzer (BZ-X710, KEYENCE).

### Time of addition analysis

VeroE6/TMPRSS2 cells were inoculated with virus at an MOI of 0.001 for 1h and the unbound virus was removed by washing. Cells were cultured for 24 h and the amount of viral RNA in the supernatant was measured as an indicator of released virus particles. Compounds were added with three different timing: (a, whole) compounds present throughout the experiment, (b, entry) compounds were treated for 1 h during viral inoculation and an additional 2 h and then removed, and (c, post-entry) compounds were added at 2 h after washing free viruses and remained throughout the experiment [56–58]. Viral RNA in culture supernatant was measured as described above.

### SARS-CoV-2 and SARS-CoV pseudovirus infection assay

SARS-CoV-2 and SARS-CoV pseudoviruses were produced using vesicular stomatitis virus (VSV)-pseudovirus system as described previously [59,60] using expression plasmids encoding SARS-CoV-2 or SARS-CoV spike protein and the G-deficient VSV which carries the luciferase gene instead of the VSV-G gene. This pseudoviruses infect the target cells in a spike-dependent manner and produce luciferase activity in infected cells. VeroE6/TMPRSS2 cells were inoculated with SARS-CoV-2 or SARS-CoV pseudovirus in the presence or absence of compounds and intracellular luciferase activity was measured at 24 h post-inoculation using Luciferase Assay System (Promega).

### Virus-cell attachment assay

VeroE6/TMPRSS2 cells were incubated with viruses in the presence or absence of compounds for 5 min (Fig 3D) or 60 min (Fig 5B) at 4°C. After washing the unbound virus, the attached viral RNA was extracted with RNeasy Mini Kit (QIAGEN) and quantified by real time RT-PCR.

### Compound treatment to SARS-CoV-2 particles or host cells

To evaluate the effect of compounds on SARS-CoV-2 particles, virus was treated with or without compounds for 60 min at 37°C, and unbound compounds were subsequently removed by ultrafiltration using centrifugal filter devices (Pall Corpotation). The viral sample ultrafiltrated without compound was used as a control. The attachment capacity of compound-treated virus was evaluated by treating VeroE6/TMPRSS2 cells in the absence of compounds for 30 min at 4°C, and then extracting the attached viral RNA with RNeasy Mini Kit (QIAGEN) for quantification by real time RT-PCR. To evaluate the effect of compounds on host cells, VeroE6/TMPRSS2 cells were pretreated with compounds for 30 min at 37°C before being washed. The capacity of compound-treated cells to virus attachment was measured by inoculating the cells with virus for 30 min at 4°C in the absence of compounds and detecting the attached viral RNA.

### The glycan array

Glycopeptide conjugated BSA was prepared by glycoconjugation of BSA using N-(3-maleimidobenzoyloxy) succinimide (Nacalai), with each glycopeptide by catalytic reaction. Preparation of the glycopeptide conjugated BSA microarray and binding assay of glycan microarray were carried out as reported previously [61,62]. The amount of glycans on the neoglycoprotein was determined by liberation of glycans, fluorescence labeling and quantified by HPLC (α2-6-SGP and asialo-SGP were 10.8 mol glycan/ 1 mol BSA and α2-3-SGP was 10.5 mol glycan/1 mol of BSA). Anti-SARS-CoV-2 S1 antibody (prepared in house) was used as a primary antibody, and Cy3-conjugated anti-rabbit IgG was used as secondary antibody. Following MIRAGE guidelines, information on the glycan array is shown.

1. Glycand binding sample and assay conditions: Recombinant SARS-CoV-2 spike S1 (Sino Biological)
2. Glycan library: α2-6-SGP, α2-3-SGP, and asialo-SGP (Fushimi Pharmaceutical) were conjugated to BSA by *m*-maleimidobenzoyl-*N*-hydroxysuccinimide ester.
3. Printing surface: Epoxy coated slide glass (Rexxam).
4. Array Printer: BioJet plus displacer AD1500 (BioDot)
5. Glycan microarray with "map": The glycan signals of triplicate spots were averaged and normalized to the mean value of 21 glycans immobilized on the array.
6. Detector and data processing: Evanescent Fluorescence Scanner Bio-REX Scan 200 (Rexxam).
7. Glycan microarray data presentation: The results are shown in Fig 4.
8. Interpretation and conclusion from microarray data: α2-6-sialoglycans were bind to recombinant SARS-CoV-2 spike S1.

### Neuraminidase treatment

To examine the role of sialic acid in virus infection, VeroE6/TMPRSS2 cells were incubated with neuraminidase derived from either *Vibrio cholerae* (Roche) or *Arthrobacter ureafaciens* (Nacalai) for 2 h at 37°C. After washing cells to remove neuraminidase, SARS-CoV-2 and SARS-CoV pseudovirus infection assay or SARS-CoV-2 attachment assay was performed as described above.

### Lectin staining

For detection of sialic acids, VeroE6/TMPRSS2 cells were fixed with 4% paraformaldehyde. Biotinylated elderberry bark lectin and biotinylated *Maackia amurensis* lectin II (Vector Labs) were used to stain α2–6 and α2–3 sialic acid, respectively. The lectins were added into fixed cells for 1 h at room temperature, then streptavidin-conjugated Alexa Fluor 594 (Thermo Fisher Scientifics) and DAPI were added for 1 h at room temperature. The fluorescence images were captured using confocal microscopy TCS SP8 (Leica).

### Co-immunoprecipitation analysis

The cells were lysed with the buffer [50 mM Tris-HCl (pH 7.4), 150 mM NaCl, 5 mM EDTA, 1% NP-40 and complete protease inhibitor], followed by an immunoprecipitation with anti-ACE2 antibody (R&D Systems) or mouse normal IgG as a negative control as described previously [63].

### Immunoblot analysis

Immunoblot analysis was performed as described previously [64] using anti-ACE2 (R&D Systems), anti-SARS-CoV-2 spike (Sino Biological) or anti-actin (Sigma) antibodies as primary antibodies. The intensity of the bands in Fig 5B was measured using ImageJ software.

### Statistics

All statistical data were obtained by triplicates of three cell culture wells in an assay. All experiments were repeated at least two times in each assay and the representative data are shown in figures. Immunoblot analysis, indirect immunofluorescence analysis and lectin staining (Figs 2B, 5B–5D, 5F-a and S3) show one representative data, however, these experiments were repeated at least two times in each assay. Statistical significance was estimated using the two-tailed Student’s t test (*p < 0.05; **p < 0.01; N.S., not significant).

## Supporting information

S1 Fig Chemical structures of sialylglyco-polyglutamic acid (PGA) and sialylglycopeptide (SGP). The structures are presented in the same formation as in Fig 1. For polymeric compounds, the estimated average molecular weights (Mw) are indicated, which have been obtained based on DS and averaged Mw of each polymer backbone. The values of *m* and *n* indicating degree of polymerization are rounded integers for average structures. Polymer compounds contain spacer groups, aminopentyl in α2-3SLN-PGA and α2-6SLN-PGA, and aminoethyl Lys in α2-6SLN-lipo-PGA, which are designed not to interfere with receptor-ligand interactions. A spacer in α2-6SLN-lipo-PGA is acylated, which has been shown to mimic sphingosine as in LS-PGA.
(PDF)

S2 Fig The viability of cells and the effect of additional compounds on SARS-CoV-2 infection. **(A)** Schematic representation of the schedule for treating VeroE6/TMPRSS2 cells with compounds and SARS-CoV-2 in the infection assay. Black and white boxes indicate periods of treatment and nontreatment, respectively. **(B, C)** Viability of cells treated with or without the compounds was measured by quantification of survived cell numbers using ImageXpress Micro Confocal (Molecular Device). S1B and S1C Fig corresponds to Fig 2A and 2C, respectively. **(D, E)** SARS-CoV-2 infection assays were performed in the presence or absence of the indicated compounds. SARS-CoV-2 or SARS-CoV infection was determined by detecting SARS-CoV-2 RNA in the culture supernatant. Remdesivir, 10 μM; 2-*O*-methyl α-D-*N*-acetylneuraminic acid (Me-α-Neu5Ac), 60 mM; 2–6 sialyllactose sodium salt, 150 mM.
(PDF)

S3 Fig The detection of sialic acid of the cells treated with or without neuraminidase. VeroE6/TMPRSS2 cells were treated with or without indicated neuraminidases for 2 h at 37°C, then cells were fixed and sialic acids were detected by lectin staining. Elderberry bark lectin for α2–6 linked sialic acid and *Maackia amurensis* lectin II for α2–3 linked sialic acid. Neuraminidase from *Arthrobacter ureafaciens*, 100 mU/ml; from *Vibrio cholerae*, 40 mU/ml; elderberry bark lectin, 20 μg/ml; *Maackia amurensis* lectin II, 20 μg/ml.
(PDF)
