# Supplementary figures and images for: Significant role of host sialylated glycans in the infection and spread of severe acute respiratory syndrome coronavirus 2
Source: PLoS Pathog. 2022 Jun 14;18(6):e1010590. doi: 10.1371/journal.ppat.1010590 (PMC9197039; doi:10.1371/journal.ppat.1010590)

**Fig. S1 Chemical structures of sialylglyco-polyglutamic acid (PGA) and sialylglycopeptide (SGP)**

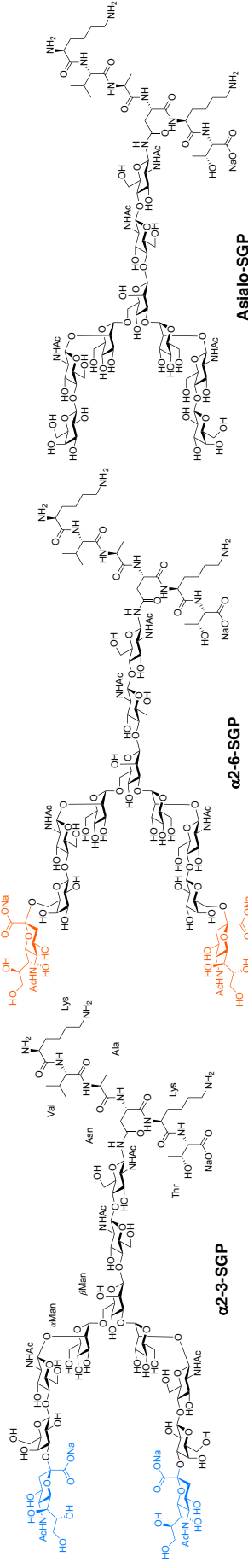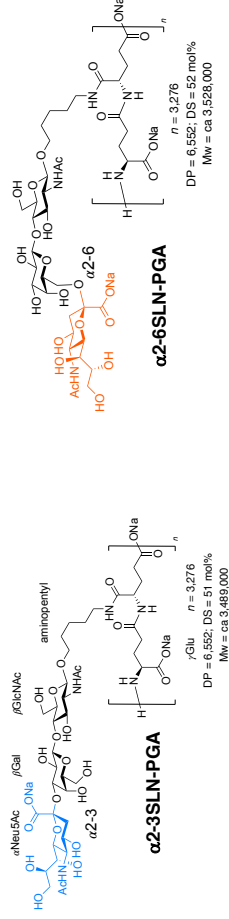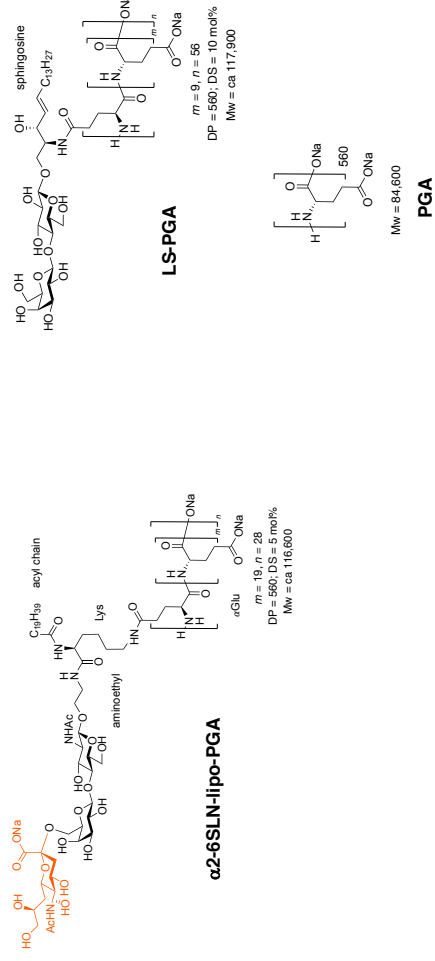

Supplement: S1 Fig — The structures are presented in the same formation as in Fig 1. For polymeric compounds, the estimated average molecular weights (Mw) are indicated, which have been obtained based on DS and averaged Mw of each polymer backbone. The values of m and n indicating degree of polymerization are rounded integers for average structures. Polymer compounds contain spacer groups, aminopentyl in α2-3SLN-PGA and α2-6SLN-PGA, and aminoethyl Lys in α2-6SLN-lipo-PGA, which are designed not to interfere with receptor-ligand interactions. A spacer in α2-6SLN-lipo-PGA is acylated, which has been shown to mimic sphingosine as in LS-PGA. (PDF) [file ppat.1010590.s001.pdf]

Fig. S2 The viability of cells and the effect of additional compounds on SARS-CoV-2 infection

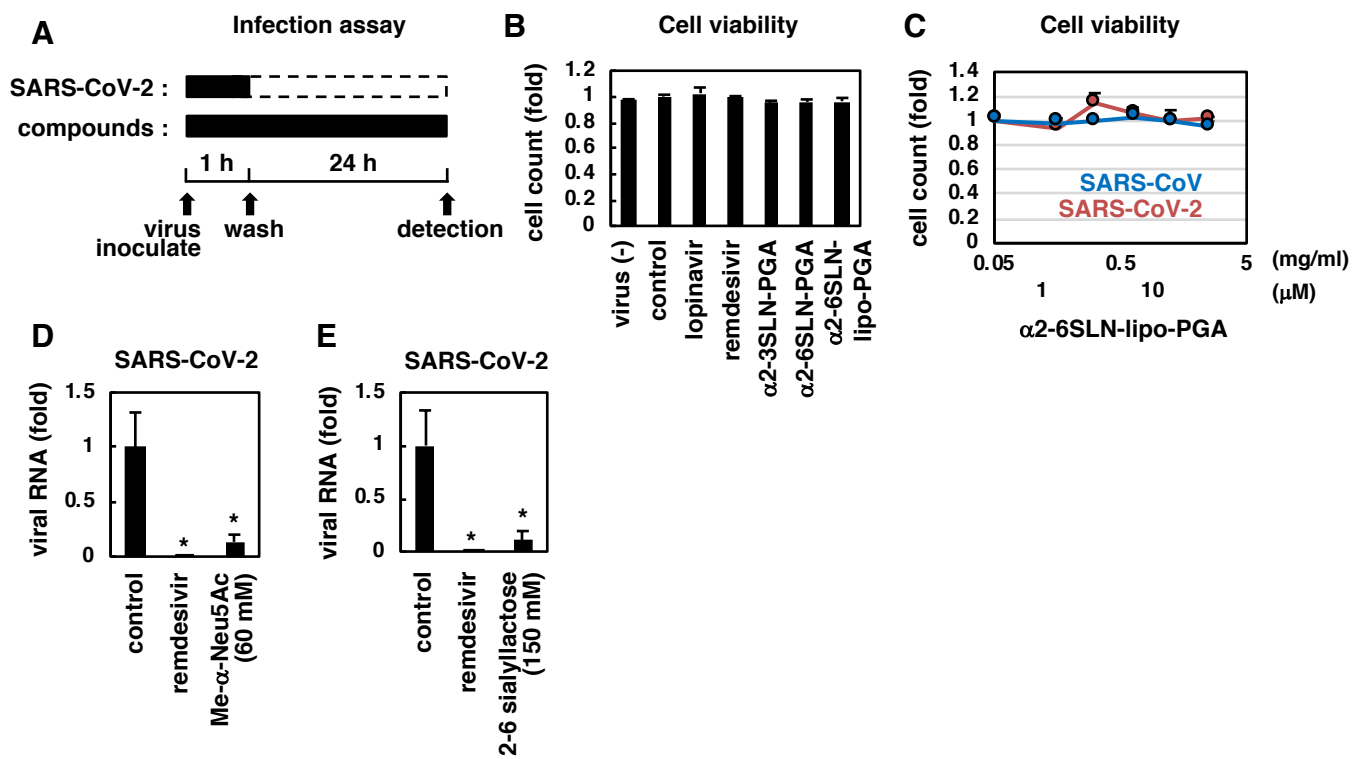

Supplement: S2 Fig — (A) Schematic representation of the schedule for treating VeroE6/TMPRSS2 cells with compounds and SARS-CoV-2 in the infection assay. Black and white boxes indicate periods of treatment and nontreatment, respectively. (B, C) Viability of cells treated with or without the compounds was measured by quantification of survived cell numbers using ImageXpress Micro Confocal (Molecular Device). S1B and S1C Fig corresponds to Fig 2A and 2C, respectively. (D, E) SARS-CoV-2 infection assays were performed in the presence or absence of the indicated compounds. SARS-CoV-2 or SARS-CoV infection was determined by detecting SARS-CoV-2 RNA in the culture supernatant. Remdesivir, 10 μM; 2-O-methyl α-D-N-acetylneuraminic acid (Me-α-Neu5Ac), 60 mM; 2–6 sialyllactose sodium salt, 150 mM. (PDF) [file ppat.1010590.s002.pdf]

Fig. S3 The detection of sialic acid of the cells treated with or without neuraminidase

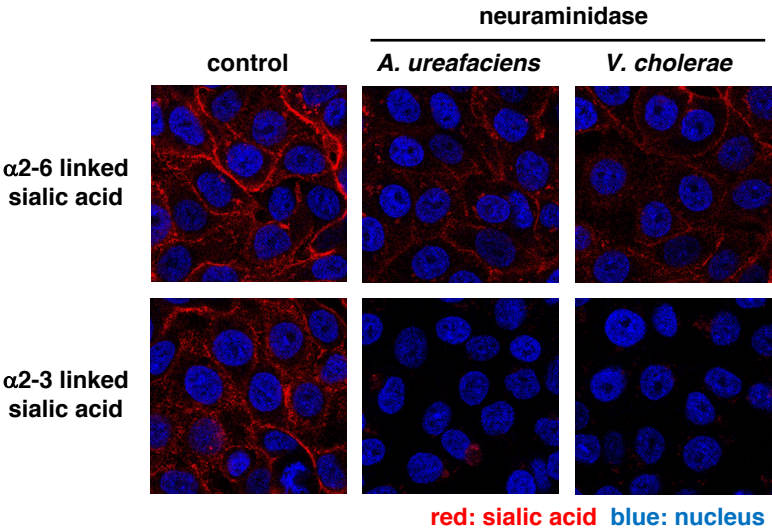

Supplement: S3 Fig — VeroE6/TMPRSS2 cells were treated with or without indicated neuraminidases for 2 h at 37°C, then cells were fixed and sialic acids were detected by lectin staining. Elderberry bark lectin for α2–6 linked sialic acid and Maackia amurensis lectin II for α2–3 linked sialic acid. Neuraminidase from Arthrobacter ureafaciens, 100 mU/ml; from Vibrio cholerae, 40 mU/ml; elderberry bark lectin, 20 μg/ml; Maackia amurensis lectin II, 20 μg/ml. (PDF) [file ppat.1010590.s003.pdf]
